# Superhydrophobic Surface-Assisted Preparation of Microspheres and Supraparticles and Their Applications

**DOI:** 10.1007/s40820-023-01284-2

**Published:** 2024-01-04

**Authors:** Mengyao Pan, Huijuan Shao, Yue Fan, Jinlong Yang, Jiaxin Liu, Zhongqian Deng, Zhenda Liu, Zhidi Chen, Jun Zhang, Kangfeng Yi, Yucai Su, Dehui Wang, Xu Deng, Fei Deng

**Affiliations:** 1 Institute of Fundamental and Frontier Sciences, University of Electronic Science and Technology of China, Chengdu, 611731 People’s Republic of China; 2 School of Materials Science and Engineering, Sun Yat-Sen University, Guangzhou, 510275 People’s Republic of China; 3 Shenzhen Institute for Advanced Study, University of Electronic Science and Technology of China, Shenzhen, 518110 People’s Republic of China; 4 Pharmaceutical Glass Co. Ltd, Zibo, 256100 People’s Republic of China; 5 Department of Nephropathy, School of Medicine, Sichuan Provincial People’s Hospital, University of Electronic Science and Technology of China, Chengdu, People’s Republic of China; 6 Department of Nephrology, Sichuan Provincial People’s Hospital Jinniu Hospital, Chengdu Jinniu District People’s Hospital, Chengdu, People’s Republic of China

**Keywords:** Superhydrophobic surface, Microspheres and supraparticles, Photonic devices, Catalysts, Biomedical and trace detections

## Abstract

Superhydrophobic surface (SHS) has been well developed, as SHS renders the property of minimizing the water/solid contact interface. Water droplets deposited onto SHS with contact angles exceeding 150°, allow them to retain spherical shapes, and the low adhesion of SHS facilitates easy droplet collection when tilting the substrate. These characteristics make SHS suitable for a wide range of applications. One particularly promising application is the fabrication of microsphere and supraparticle materials. SHS offers a distinct advantage as a universal platform capable of providing customized services for a variety of microspheres and supraparticles. In this review, an overview of the strategies for fabricating microspheres and supraparticles with the aid of SHS, including cross-linking process, polymer melting, and droplet template evaporation methods, is first presented. Then, the applications of microspheres and supraparticles formed onto SHS are discussed in detail, for example, fabricating photonic devices with controllable structures and tunable structural colors, acting as catalysts with emerging or synergetic properties, being integrated into the biomedical field to construct the devices with different medicinal purposes, being utilized for inducing protein crystallization and detecting trace amounts of analytes. Finally, the perspective on future developments involved with this research field is given, along with some obstacles and opportunities.

## Highlights

- An overview of the superhydrophobic surface -assisted strategies for fabricating microspheres and supraparticles are presented.
- The applications of microspheres and supraparticles fabricated using SHS-assisted strategies are discussed in detail.
- NCrucial challenges facing the development of microspheres and supraparticles fabricated through SHS-assisted strategies are analysed.

## Graphical abstract

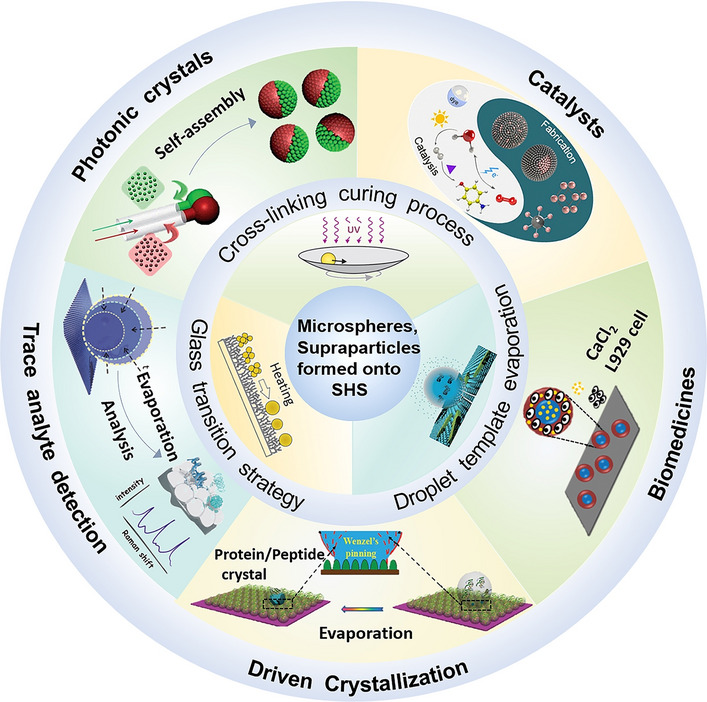

## Introduction

Wettability, typically defined as the tendency of a liquid to spread out on a solid, has been one of the most important characteristics of a solid surface. The superhydrophobic surface (SHS) represents a specific solid surface on which a droplet of water shows a contact angle greater than 150°, thus manifesting excellent water repellency. It is found that biological surfaces, such as those of certain plants and animals, exhibit excellent superhydrophobic capabilities due to their suitable morphologies and specific surface chemistry properties [1–3]. Inspired by nature, superhydrophobicity has been successfully mimicked through a rational design of surface roughness and proper regulation of surface energy [4–6]. The extensive design and fabrication of artificial SHS have driven the potential development in many fields, such as self-cleaning [5, 7–9], anti-fogging [10–14], anti-icing [15–19], oil/water separation [20–23], water collection [24–27], liquid transportation [28–34], anti-corrosion [35–41] and anti-fouling [42–47].

SHS with low adhesion is one of the most common types of SHS [29, 48–50]. It has a large contact angle (more than 150°), as well as a low sliding angle (less than 10°) [51–53]. The water drop standing on this surface acts like a sphere and it can easily roll off from the surface, allowing the droplet to be well collected [54–56]. Based on this phenomenon, a range of functional materials, particularly microspheres and supraparticles, have been successfully fabricated. Microspheres refer to individual tiny spherical particles, with typical diameters in the micrometer range was 1–1,000 μm [57–59]. Supraparticle is a term to describe a type of three-dimensional macroscopic structure formed by dispersed nano- or microparticles through self-assembly, typically ranging in size from a few 10 μm to several 100 μm [60, 61]. The microspheres are typically obtained on low-adhesion SHS through cross-linking curing reactions [62, 63], polymer melting method [64] and droplet template evaporation strategy [65, 66]. Supraparticles are formed by evaporating droplet templates on SHS [67]. These SHS-assisted fabrication strategies offer several advantages, including material saving, reduced organic pollution, and high throughput production, aligning with the principles of environmental protection, cost-effectiveness, and energy efficiency [63, 68]. Moreover, through these strategies, microspheres and supraparticles with controllable morphologies, customizable structures and tunable properties can be easily acquired by adjusting the formula and properties of droplets. These technical superiorities make SHS popular for fabricating supraparticles and microspheres. It is worth mentioning that the superamphiphobic surface (SAS), serving as a special type of SHS, showcases a unique combination of essential characteristics. In addition to possessing the requisite surface roughness and low surface energy characteristic of SHS, SAS also boasts specific topographical features, including overhangs, reentrant geometries, or convex curvatures [69]. This unique configuration imparts remarkable repellent properties, not only against aqueous substances but also towards oily liquids such as hexadecane, ethylene glycol, and tetradecane. This versatility enables SAS to be utilized in the fabrication of microspheres and supraparticles from various liquid droplets, extending beyond the confinement of water droplets.

In this review, a comprehensive summary of the fabrication strategies assisted by SHS is provided first. These strategies can be broadly classified into three distinct categories: cross-linking curing, polymer melting, and droplet template evaporation methods. Each category will be discussed in detail. Especially, in the droplet template evaporation method, the factors that regulate the morphologies, structures, and properties of supraparticles will be introduced in detail. Subsequently, the devices consisting of microspheres and supraparticles are summarized according to their applications in colloidal photonic crystals, catalysts, biological medicine, protein crystallization, and trace analyte detection. Finally, the development prospects and remaining challenges of this research field are presented.

## SHS-Assisted Fabrication Strategies for Versatile Microspheres and Supraparticles

The fabrications of microspheres and supraparticles take advantage of the high liquid repellency and low adhesion of SHS. These SHS-assisted fabrication strategies can be divided into three categories, including cross-linking curing method, polymer melting process, and droplet template evaporation strategy. It is worth mentioning that only liquid droplets with the appropriate properties and suitable size ranges can remain spherical on SHS [70, 71].

### Cross-linking Curing Strategy

**Fig. 1:**
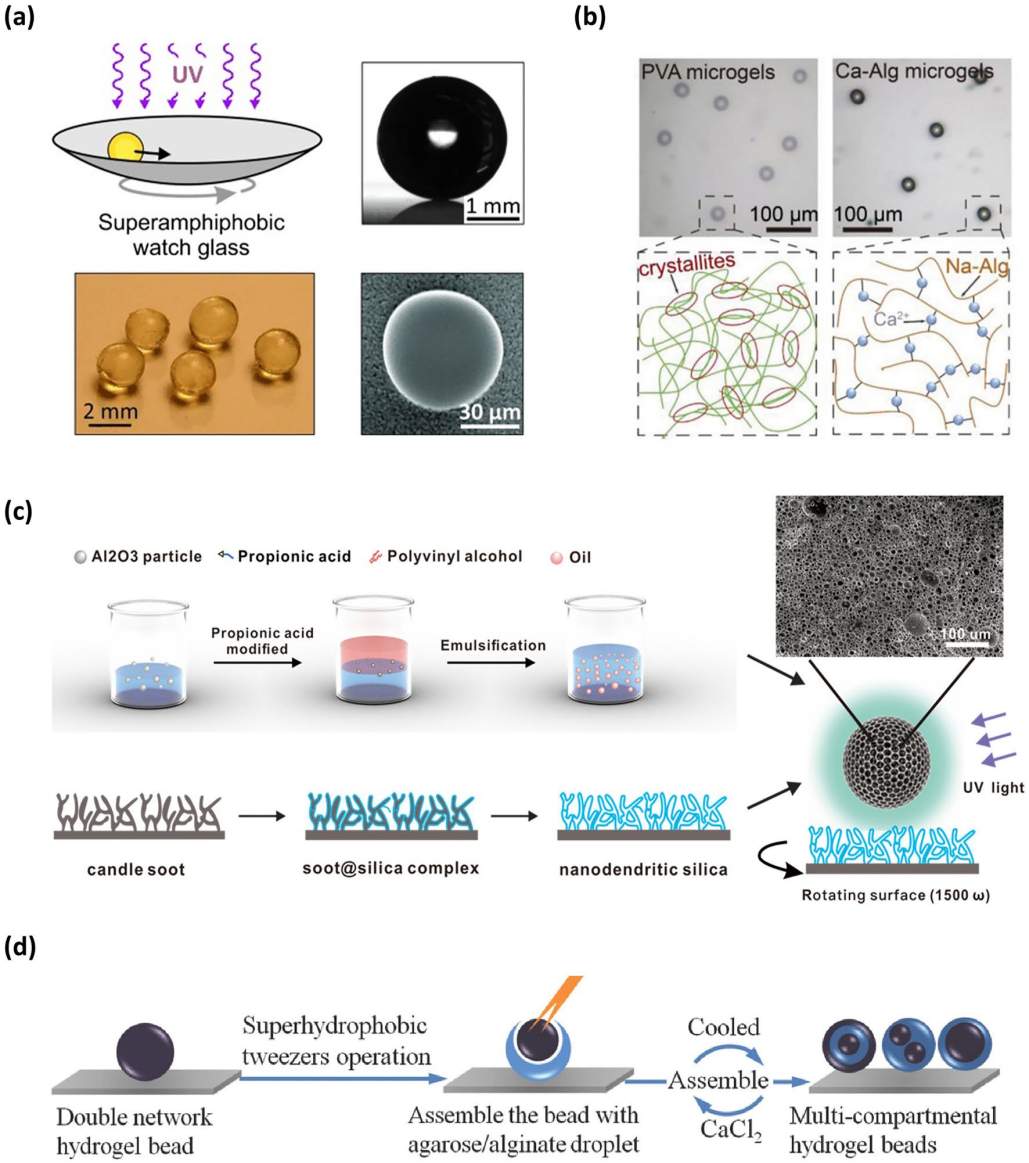
Microsphere fabricated by SHS-assisted cross-linking or polymerization strategy. **a** Polymer microsphere obtained onto the SAS via UV-induced radical polymerization reaction. Reproduced with permission from Ref. [64]. Copyright 2013, Wiley–VCH. **b** PVA and ALG hydrogel microsphere formed via a cross-linking reaction. Reproduced with permission from Ref. [65]. Copyright 2021, Wiley–VCH. **c** Porous Al_2_O_3_ microspheres obtained onto SAS via a UV-induced cross-linking reaction. Reproduced with permission from Ref. [83]. Copyright 2021, Elsevier. **d** Multi-compartmental microsphere fabricated onto SHS via sequential polymerization or cross-linking process. Reproduced with permission from Ref. [85]. Copyright 2014, Wiley–VCH

SHS-assisted fabrication strategies for preparing the microspheres rely on three types of reactions. One is a cross-linking or polymerization reaction. When the liquid droplets containing polymerizable monomers or cross-linkable polymers are placed onto SHS, the liquid droplets acquire spherical shapes. With the aid of mild conditions (e.g., UV-irradiation [64], target ions addition [72], pH change [73] and temperature modulation [74]), the monomers or polymers appear in-situ polymerization or cross-linking reaction, which causes the hardening of these spherical droplets, thus forming solid or hydrogel microspheres onto SHS [62–65, 72–76]. Due to the low adhesion of SHS [77], the resulting microspheres can be easily collected by tilting the SHS. For instance, Hans-Jürgen Butt’s group used an in-situ cross-linking method initiated by UV light to obtain polymer microspheres [64]. In this approach, the droplet composed of glycerolate dimethacrylate and tri(ethylene glycol) dimethacrylate, along with a photo-initiator, was deposited on SAS and formed a spherical shape. After UV light irradiation, the methacrylate in the droplet underwent radical polymerization, resulting in the hardening of the droplet and the formation of the polymer microsphere (Fig. 1a). To avoid deformation of the droplet during the polymerization process, the group designed a bowl-shaped SAS and continuously moved the surface to keep the droplets in a rolling motion state.

Apart from the polymer microspheres mentioned above, the SHS-assisted cross-linking strategy can also be utilized to fabricate various hydrogel microspheres [62, 63, 74, 76]. For instance, by dispensing droplets of alginate (ALG) solution on SHS and subsequently adding Ca^2+^ to the system, spherical ALG hydrogel microspheres can be formed on SHS due to the Ca^2+^-induced cross-linking of ALG (Fig. 1b) [65, 78, 79]. By repeating freezing and melting operations, Polyvinyl alcohol (PVA) hydrogel microspheres can be formed onto SHS due to the formation of physical cross-linking networks (Fig. 1b) [65, 80, 81]. By adjusting the pH value of the solution or adding polyvalent anions, spherical droplets of chitosan solution formed onto SHS can transform into hydrated microspheres, as these conditions cause cross-linking between chitosan molecules [73, 82].

Moreover, microspheres with specific functions and structures can also be easily achieved via this method. By introducing magnetic Fe_3_O_4_ particles into a hydrogel precursor solution and dispersing the droplets of the combined solution onto the SHS, followed by a subsequent cross-linking process, the hydrogel microspheres rapidly exhibited magnetic responsiveness [65]. By incorporating CdTe quantum dots into the precursor solution, the produced microspheres via a SHS-assisted cross-linking strategy can obtain fluorescent properties [65]. Moreover, by incorporating inorganic Al_2_O_3_ particles into the organic PVA precursor solution to form an Al_2_O_3_-PVA composite microgels onto SAS and then using organic phase as sacrificial templates, a pure inorganic Al_2_O_3_ microsphere with hierarchical porous structure was obtained (Fig. 1c) [83, 84]. Furthermore, relying on sequential polymerization or cross-linking process, the multi-compartmental microspheres, such as core–shell structure, multi-layer structure and multi-core structure, can be realized onto a SAS (Fig. 1d) [72, 85, 86].

### Polymer Melting Method

**Fig. 2:**
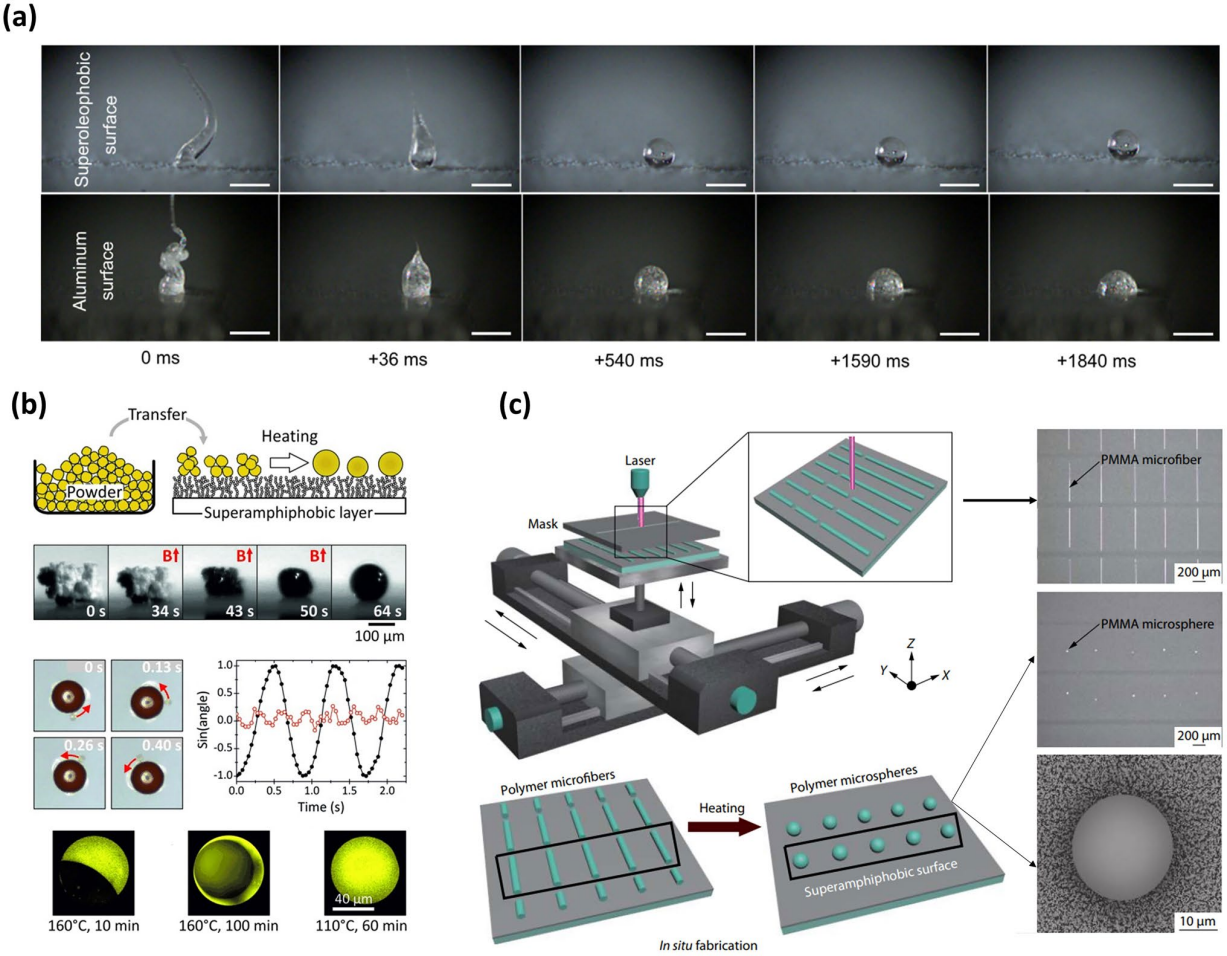
Microspheres fabricated by SHS-assisted polymer melting process. **a** Polymer melts onto a non-wetting surface and a wetting surface captured by a high-speed camera, where they only form ball shapes onto the non-wetting surface. Reproduced with permission from Ref. [87]. Copyright 2018, Elsevier. **b** Magnetic and Janus microspheres obtained onto SAS by heating the polymer powder to a visco-elastic state and then cooling it. Reproduced with permission from Ref. [64]. Copyright 2013, Wiley–VCH. **c** Schematic illustration of large quantities of uniform microspheres produced onto SAS using microfiber processing via a polymer melting strategy. Reproduced with permission from Ref. [88]. Copyright 2020, Springer

The polymer melting method is an additional strategy for preparing microspheres, wherein polymer powders are heated above the polymer melting temperature on SHS or SAS and then cooled using natural air. Due to the high liquid repellency of SHS or SAS, these polymer melts form spherical droplets on these surfaces during the heating process. Upon cooling, the melts undergo a phase transition and solidify into polymer microspheres (Fig. 2a) [87]. Recently, with the help of this SHS-assisted polymer melting strategy, a variety of microspheres have been easily fabricated by simply replacing the powder composition (Fig. 2b) [64].

Additionally, by incorporating different polymer types or adding functional substances, functional microspheres such as Janus microspheres and magnetic microspheres can be synthesized [64]. It should be noted that the formation of Janus microspheres composed of two components is also closely related to annealing conditions, as the annealing process significantly affects the degree of phase separation. As reported by Hans-Jürgen Butt’s group, the polystyrene (PS)/poly(methyl methacrylate) (PMMA) Janus microspheres were formed onto SAS at an annealing condition of 160 °C for 10 min. With the increase of annealing time (160 °C for 100 min), PS was completely embedded PMMA microsphere. Furthermore, when the annealing condition was set to 110 °C for 60 min, a uniform PS-PMMA microsphere was formed onto SAS without phase separation. Generally, this polymer melting strategy offers a flexible approach for synthesizing one-component or multi-component microspheres. However, there is a great challenge in the synthesis of continuously tunable and uniform microspheres. To overcome this limitation, the concept of utilizing in-fiber fluid instability has been proposed to achieve scalable microparticles [88]. In this approach, an ordered microfiber array was first formed on SAS at room temperature, and then each microfiber broke up into uniformly sized spherical droplets when annealed above the viscous flow temperature. After cooling, these spherical droplets solidified into polymer microspheres (Fig. 2c).

### Droplet Template Evaporation Strategy

#### Droplet Template Evaporation Strategy for Constructing Microspheres

Several microspheres have been produced using the SHS-assisted droplet template evaporation strategy [65, 66, 89]. In this manner, the target substance is initially dissolved in a solvent to form a homogeneous solution, and then the droplets of the solution are deposited onto SHS. The interfacial tension induces the droplet to be shaped into a spherical morphology. As the solvent evaporates, the three-phase contact line (TPCL) of the droplet slides freely over the SHS at a constant receding contact angle, resulting in decreasing droplet size and unchanged droplet shape, ultimately forming a hardened microsphere. For example, Poly(D, L-lactide-co-glycolide) (PLGA) microspheres have been successfully fabricated by placing drops of dioxane solutions containing PLGA onto SAS and allowing the solvents to evaporate [66]. This developed polymer- and solvent-independent approach allows a variety of microspheres to be formed onto SHS or SAS. Additionally, by employing a specialized setup that allows simultaneous feeding of multiple raw material solutions, microspheres with unique structures can be prepared. For example, Deng’s group utilized a microfluidic spinning instrument with a coaxial needle to print two raw material solutions onto SHS, generating the solution columns [65]. Due to the Plateau-Rayleigh instability of a liquid jet [90–92] and the high liquid repellency of SHS [93–95], these solution columns broke up into uniformly spherical droplets at ambient temperature. After the solvents in the inner core and outer cladding structure evaporated, core–shell microspheres were obtained [65]. The introduction of this device enables the high-throughput production of various core–shell microspheres.

**Fig. 3:**
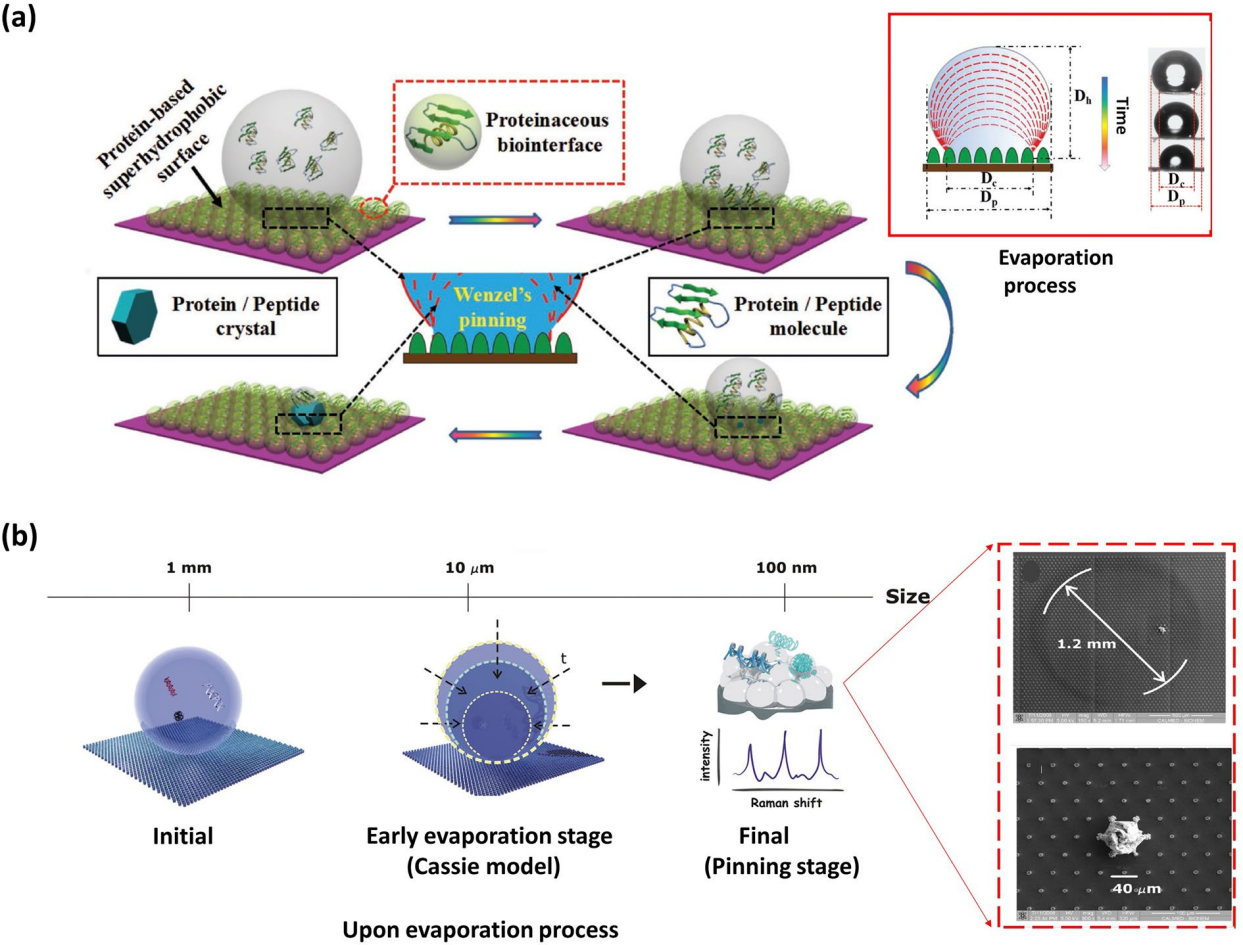
Microsphere-like materials defined in the review showing various shapes fabricated by SHS-assisted droplet template evaporation strategy.** a** Schematic illustration of the formation of prismatic-shaped crystalline materials on a protein-based SHS, where the inset in a red solid frame describes the morphologic change of the droplet onto the SHS during the evaporation process. Reproduced with permission from Ref. [96]. Copyright 2018, Wiley–VCH. **b** Schematic illustration to describe the formation of shapeless microsphere materials via a droplet evaporation process onto SHS, where the spherical droplet first follows a Cassie model and subsequently undergoes an irreversible transition to a pinning state, resulting in the concentration of solutes into an ultra-small region. The inset in the red dotted box shows SEM images of concentrated solute under different magnifications. Reproduced with permission from Ref. [98]. Copyright 2012, American Chemical Society

Notably, in the SHS-assisted droplet template evaporation strategy, some materials may not form spheres, as the drops are pinned and thus cannot move freely during the evaporation process. In this review, these materials are classified as microsphere-like materials because their formation process resembles that of microspheres. Yang and colleagues reported the formation of a prismatic-shaped crystalline material on a protein-based SHS (Fig. 3a) [96]. In this case, the droplets of protein buffer were deposited on the SHS in the form of balls. As the water content continuously evaporated, the droplets were pinned on SHS. The pinning effect caused a stable contact diameter (Dc) during evaporation, while the projection diameter (Dp), droplet height (Dh), and contact angle all decreased over time (the inset in Fig. 3a) [96]. The pinning state observed in droplets can be attributed to two factors. One is protein buffer has a lower surface tension than water [96]. Another is the protein-based SHS possesses inherent multiple polar residues that may enhance the adhesion between SHS and droplets [96, 97]. This pinning phenomenon leads to the formation of a prismatic shape, instead of a spherical shape [96, 97]. Additionally, shapeless materials can also be formed using this strategy. When a droplet extracted from a diluted solution was deposited on an abiotic SHS, it initially followed the Cassie model and moved freely over the SHS during the evaporation process. Subsequently, an irreversible transition from the Cassie to Wenzel state occurred, resulting in the collapse of the droplet’s center and the formation of shapeless materials (Fig. 3b) [98].

In general, the appearance of various forms can be attributed to the difference in the dynamic TPCL behavior of spherical droplets during evaporation. The formation of ideal spheres is attributed to the uniform and continuous recession of TPCL throughout the entire evaporation process. The reason for the formation of prismatic microsphere-like materials is that the TPCL begins to pin at the initial evaporation stage and remains pinned until the completion of evaporation (the inset in Fig. 3a, framed by a red rectangle) [96]. During the evaporation process, if the TPCL recedes uniformly and continuously, and suddenly occurs a transition from a depinning to pinning state, the shapeless microsphere-like materials will be generated onto SHS (the inset in Fig. 3b) [98]. Furthermore, the cause of pinning is that the evaporation-induced driving force cannot overcome the static friction between the liquid droplets and the surface, while the reason for de-pinning is the opposite. The magnitude of static friction mainly depended on the surface type and liquid droplet properties. Consequently, the shapes of microspheres can be directly tailored by utilizing different surface types [99–101] and employing liquid droplets with varying properties.

#### Droplet Template Evaporation Strategy for Constructing Supraparticles

**Fig. 4:**
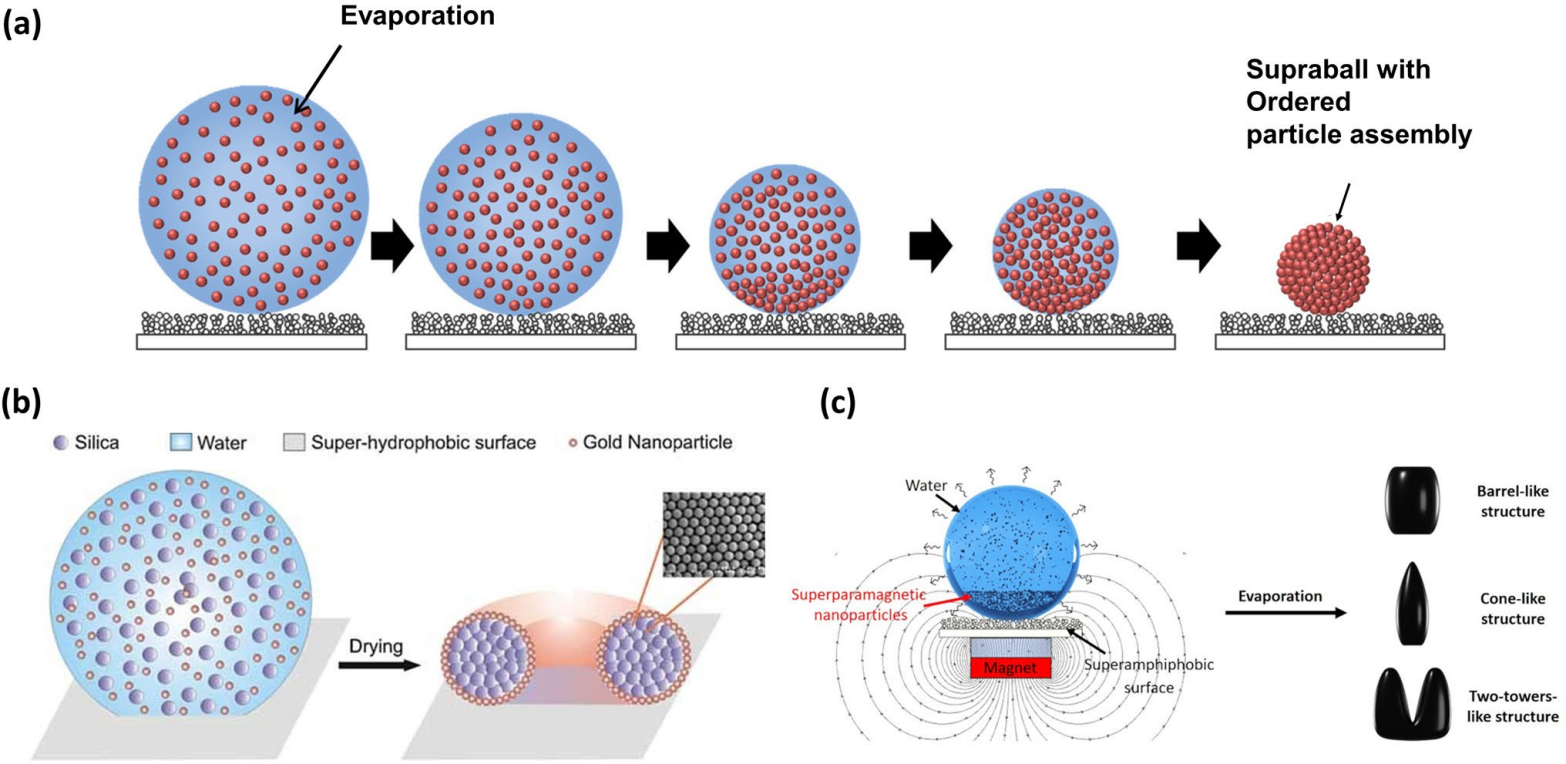
Schematic illustration of the formation of the supraparticles via a SHS-assisted droplet template evaporation strategy. **a** Spherical supraparticle. Reproduced with permission from Ref. [102]. Copyright 2018, Wiley–VCH. **b** Core–shell structured supraparticles. Reproduced with permission from Ref. [103]. Copyright 2010, Wiley–VCH. **c** Symmetric non-spherical supraparticles. Reproduced with permission from Ref. [104]. Copyright 2019, American Chemical Society

The SHS-assisted droplet template evaporation strategy has also emerged as a popular technique for supraparticle fabrication. In this approach, suspended droplets on SHS serve as templates, with each nanoparticle (NP) or microparticle approaching during the evaporation process, resulting in the self-assembly of the particles, causing a formation of supraparticles (Fig. 4a-c). This method is well-suited for the fabrication of supraparticle materials with controllable size, customizable morphology (Fig. 4a-c), tunable structure, and tailored performance, which will be described in detail below.

##### Fabricating Supraparticles with Controllable Sizes

The size of the supraparticles primarily depends on the volume of the original droplets and the concentration of NPs within them [67]. By manipulating these two factors, it is possible to generate supraparticles with diameters ranging from a few microns to sub-millimeters [67, 102, 105–107]. The upper boundary of size is dictated by gravitational forces, which, in the case of drops exceeding the capillary length, will result in drop flattening. Conversely, the fundamental lower constraint depends on the inter-protrusion spacing of rough SHS, since particles smaller than this spacing are capable of permeating SHS and causing it to be wet [64].

##### Constructing Supraparticles with Diverse Morphologies

**Fig. 5:**
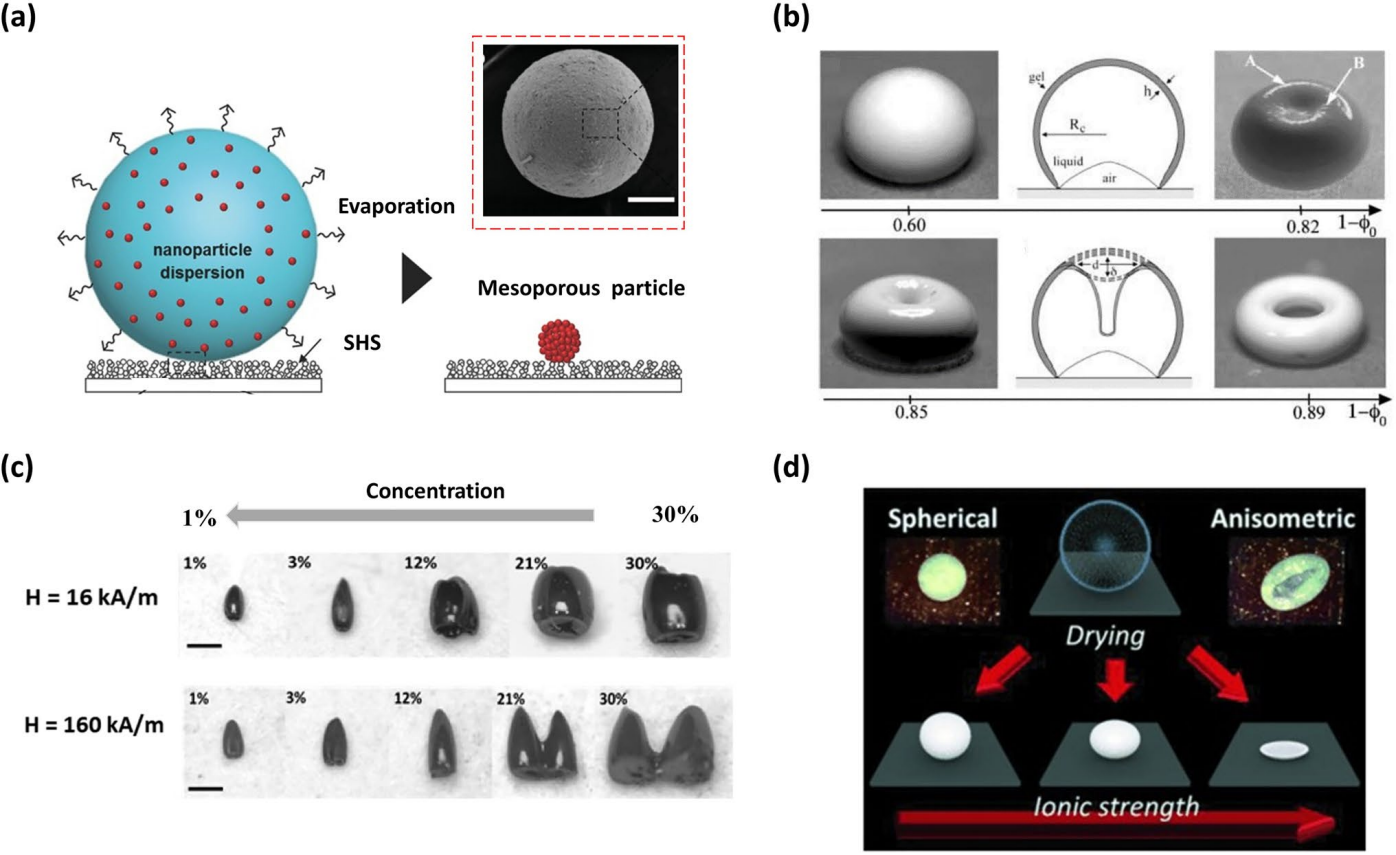
Supraparticles with various morphologies fabricated by SHS-assisted droplet template evaporation strategy. **a** Supraparticle with a spherical morphology formed on the SHS, where the inset shows the SEM image of the resulting supraparticle. Reproduced with permission from Ref. [67]. Copyright 2015, Wiley–VCH. **b** The images displaying the diverse forms produced at various values of the initial colloidal suspension concentration φ_0_, where the sketches depict the profile map of supraparticles. Reproduced with permission from Ref. [109]. Copyright 2004, IOP Publishing Ltd. **c** Optical photos of barrel-like, cone-like, and two-tower-like supraparticles prepared by using colloidal suspensions with varied concentrations (1%, 3%, 12%, 21%, 30%) and magnetic fields (16 and 160 KA m^−1^). Reproduced with permission from Ref. [104]. Copyright 2019, American Chemical Society. **d** The schematic diagram showing spherical, anisometric ellipsoidal and boat-like supraparticles obtained under different ionic strengths. The insets show the photo of spherical and anisometric boat-like supraparticle, respectively. Reproduced with permission from Ref. [115]. Copyright 2014, Wiley–VCH

The morphology of supraparticles is mainly governed by the initial morphology of droplets and the dynamic behavior of the TPCL) during the evaporation process [68]. These two factors, in turn, typically depend on the surface utilized and the characteristics of the NP suspension. Employing appropriately designed surfaces is one of the methods to achieve diverse supraparticle morphologies [99–101], but this study focuses solely on low-adhesion SHS. When a droplet of NP suspension is deposited on SHS, it initially maintains a spherical shape with a contact angle of more than 150°. Consequently, the motion behavior of TPCL is regarded as the crucial factor in determining the morphologies of supraparticles. In general, the TPCL freely moves over the SHS during evaporation, forming a spherical supraparticle (Fig. 5a) [67, 102, 105–107]. Substituting NPs with different types of colloids, such as nano-cubes, nano-plates, and nano-sheets, has no effect on supraparticle morphology [108].

Numerous approaches have been reported to regulate the dynamic behavior of TPCL for the purpose of morphology manipulation, which can be broadly categorized into two main categories. One approach involves altering the properties of NP suspensions, while the other involves introducing magnetic components into suspensions and applying an external magnetic field. For example, when adjusting the concentration of the original NP suspension to a low critical value, the TPCL remains continuously receding at the beginning, leading to a reduction in droplet size. With the further extension of evaporation time, the NPs become confined in the vicinity of the TPCL, resulting in TPCL pinning. In the final phase of evaporation, there is a notable decrease in the height of the droplet in relation to the meridian radius, causing a curvature at the center. This phenomenon becomes more pronounced as the concentration decreases (Fig. 5b) [109]. Therefore, as the concentration continuously decreases below the threshold, the in-folded or concave sphere [103, 109]、doughnut-like [103, 110], or even coffee ring [103, 111] shaped supraparticles can be realized successively. By introducing sucrose into a colloidal dispersion with varying concentration, the supraparticle morphology can be further altered, resulting in a series of new forms on SHS, such as a three-quarter sphere with a dimpled bottom, a three-quarter sphere with a flat bottom, a bagel shape with a dimpled bottom, and a pizza shape with a dimpled bottom [112]. The emergence of these morphologies is due to sucrose acting as a shape-preservative during evaporation. Additionally, by utilizing suspensions containing NPs with specific forms and properties, such as CdSe/CdS nanorod suspension or 26 vol% alumina powder suspension, a hollow dome-shaped structure, also known as an inverted bowl shape, were successfully fabricated onto SHS [113, 114]. The formation of a hollow dome-shape can be attributed to the fact that the TPCL of the drop gets pinned and increased solute concentration induces a residue solidification, while the air pockets below the droplet remain throughout the evaporation process.

By adjusting both the concentration of superparamagnetic NPs in the suspension and the intensity of the applied magnetic field, various supraparticle morphologies such as barrel-like, cone-like, and two-tower-like structures were achieved on the SHS (Fig. 5c) [104].

Furthermore, anisometric-shaped supraparticles can also be produced by modifying the specific properties of the original NP solution, such as ion strength [115–117], NP density [108], NP mono-dispersity [102, 108], and even adding volatile solvent composition [118]. For example, anisometric boat-like and ellipsoidal supraparticles can be obtained by adding salt (e.g., NaCl) beyond a threshold into fumed silica NP suspensions and subsequently drying the suspension droplets on a flat or curved V-shaped SHS (Fig. 5d) [115–117].

In summary, the dynamic behaviors of the TPCL during the evaporation process have the following effects on droplet morphology: continuous and homogeneous receding of the TPCL promotes the formation of spherical or nearly spherical supraparticles, while the pinning of the TPCL during evaporation tends to result in symmetrical non-spherical structures. Asymmetric receding of the TPCL, with pinning in one direction and homogeneous receding in the perpendicular direction, facilitates the formation of anisotropic-shaped supraparticles [119].

##### Creating Supraparticles with Various Structures

**Fig. 6:**
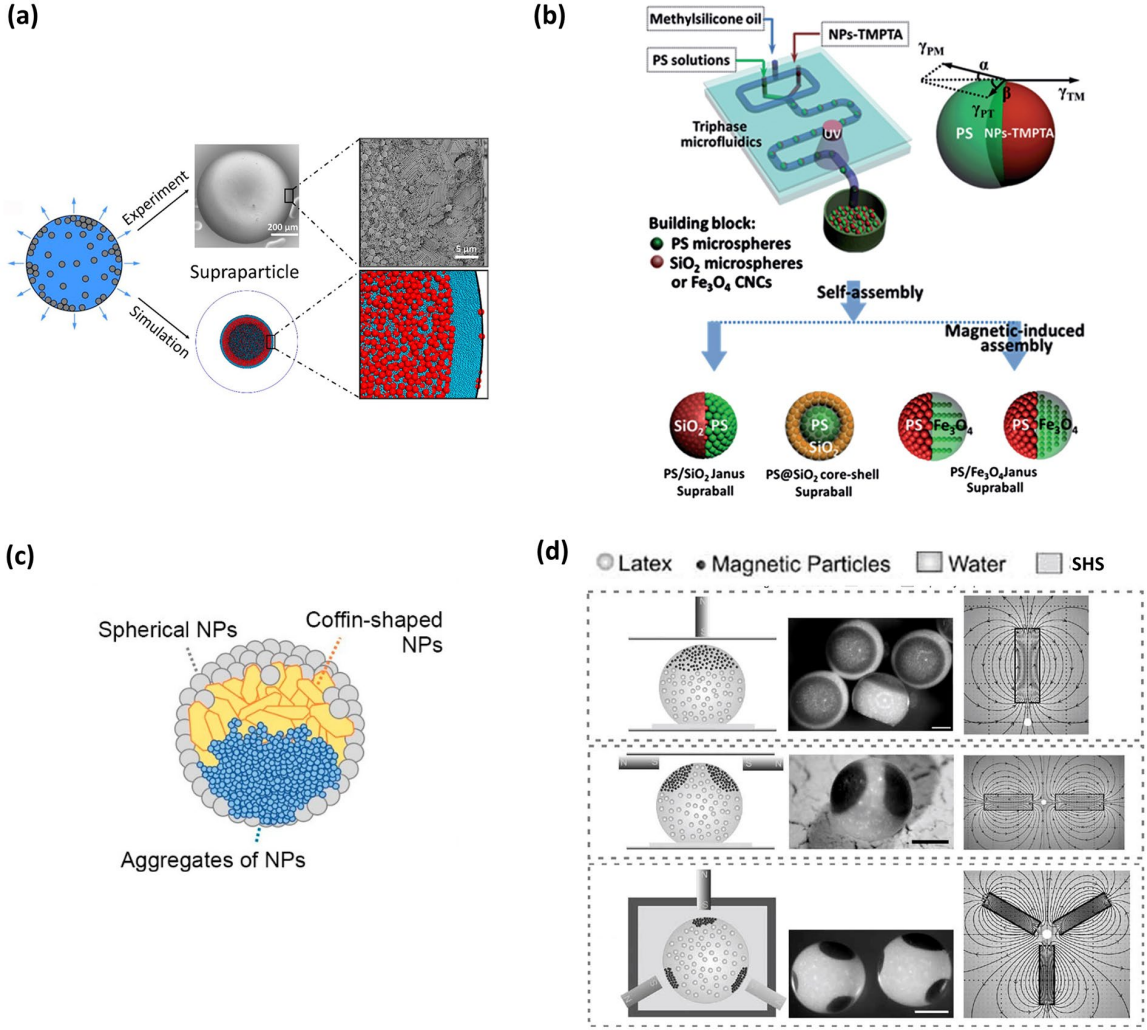
Supraparticles with various structures fabricated by SHS-assisted droplet template evaporation strategy.** a** A novel hierarchical mesoporous structure fabricated from the droplets of bi-disperse colloidal suspensions during evaporation, where mostly large colloidal NPs are found in the inner layer and small NPs are mostly distributed in the periphery. Reproduced with permission from Ref. [123]. Copyright 2019, American Chemical Society. **b** Schematic diagram of supraparticles with core–shell structure, Janus structure prepared through the utilization of a three-phase microfluidic device. Reproduced with permission from Ref. [107]. Copyright 2014, The Royal Society of Chemistry. **c** A schematic of the supraparticle with a Janus core–shell structure, where the tri-composite supraparticle consists of NP aggregates, coffin-shaped NPs and spherical NPs. Reproduced with permission from Ref. [108]. Copyright 2021, MDPI. **d** Single patch, bi-patch and tri-patch magnetic supraparticles formed under the corresponding applied electric field. The scale bars are 500 mm. Reproduced with permission from Ref. [103], Copyright 2010, Wiley–VCH

Supraparticles with diverse structures can be obtained by SHS-assisted droplet template evaporation strategy. All supraparticles show mesoporous structures since they are formed by an aggregation of NPs [67, 120–122]. By the utilization of bi-disperse colloidal suspensions, a segregation phenomenon of the small and large colloids occurs, forming a novel hierarchical mesoporous structure (Fig. 6a) [123]. The cause of the segregation is that the evaporation process results in a local increase in colloid concentration near the membrane-air interface, which in turn translates into a potential chemical gradient for the two colloids [123]. The generation of core–shell structured supraparticles can generally be achieved through two paths. One involves sequentially drying two types of colloidal dispersions onto SHS [67]. In this method, a shell is formed by adding one type of dispersion droplet onto an existing supraparticle resulting from the other dispersion and then evaporating it. An alternative is to directly produce core–shell structured droplets onto SHS using a three-phase microfluidic device with two inner cylindrical needles, followed by solvent evaporation on the SHS (Fig. 6b) [107]. In this case, the formation of a core–shell structure takes advantage of the matching surface tension and viscosity between the two immiscible colloidal suspensions. Supraparticles endowed Janus structure can also be realized by means of this microfluidic device, in which one of the inner steel needles is injected with an aqueous suspension containing one type of NPs and surfactant, while the other is injected with an organic suspension comprising another type of NPs [107]. When the continuous oil phase in the device breaks the inner solutions composed of the two kinds of suspensions at the tip of the pair of needles, uniform Janus droplets can be acquired (Fig. 6b). After evaporating the solvent of the droplets onto SHS, the Janus structures are successfully constructed. Moreover, a special Janus core–shell structured supraparticle has also been reported [108]. The structures are produced by drying liquid droplets containing spherical silica NPs, coffin-shaped zeolite, and TiO_2_ NP aggregations onto SHS (Fig. 6c) [108]. Due to the differences in density and morphology of the NPs, the resulting supraparticle shell is entirely composed of spherical NPs, while the supraparticle core exhibits a spherical Janus structure. The top half of the supraparticle core consists of coffin-shaped zeolite, while the rest is made up of TiO_2_ NP aggregations (Fig. 6c). Furthermore, by introducing superparamagnetic NPs into a colloid suspension, interesting patch structures can be formed [103]. For instance, Velev and co-workers created a series of patchy structures by adding a magnetic Iron-Nickel alloy (Fe 55%, Ni 45%) NPs into a sulfate-stabilized polystyrene latex and drying the droplets containing the mixture of latex and magnetic NPs onto SHS. Through the manipulation of magnetic fields with various spatial distributions, magnetic NPs aggregation occurred in a region of a droplet subjected to strong magnetic fields, eventually causing single, bilateral, trilateral, or even patched structures onto SHS during the evaporation process (Fig. 6d) [103].

##### Designing Supraparticles with Specific Properties

Supraparticle properties are determined by their structures and compositions. For instance, the supraparticles with high porosity generally possess excellent catalytic properties, as high porosity facilitates catalyst/adsorbate loading and enables high mass transfer flux [105]. When their building blocks are arranged in an orderly manner to form a photonic crystal structure, supraparticles can display photonic bandgap properties and bright structural colors. Undoubtedly, adjusting the composition of the NPs can also be employed to manipulate supraparticle properties. By incorporating superparamagnetic NPs into a colloidal suspension system, the supraparticle can acquire a magnetic-field response property [103]. Furthermore, Supraparticles formed by CdSe/CdS nanorods can preserve the photoluminescence properties of the pristine material [113]. Moreover, by simultaneously constructing a distinct structure and regulating the characteristics of the building blocks, supraparticles will obtain multiple functions. For example, Chen’s group reported the production of PS/Fe_3_O_4_ Janus supraparticles using the SHS-assisted droplet template evaporation strategy, where the PS and Fe_3_O_4_ hemispheres were constructed through the ordered self-assembly of monodispersed PS NPs and superparamagnetic Fe_3_O_4_ NPs, respectively. This PS/Fe_3_O_4_ Janus supraparticle exhibited both intrinsic photonic band gap property and magnetic-field response characteristics [107].

## Applications of Supraparticles and Microspheres Fabricated by SHS-Assisted Strategies

Supraparticles represent an emerging class of materials with tunable properties. Their size, shape, structure and properties can be designed by using a colloidal formula with proper properties and droplets with appropriate diameter, making them versatile in a wide range of applications, such as photonic crystals and catalysts. Microspheres are crucial in various fields due to their uniform size and high surface area. They serve as ideal carriers for high-volume encapsulation of cells and precise control of drug release. Microsphere-like materials are usually obtained during the SHS-driven protein crystallization and SHS-assisted trace solute detection processes.

### Colloidal Photonic Crystals

**Fig. 7:**
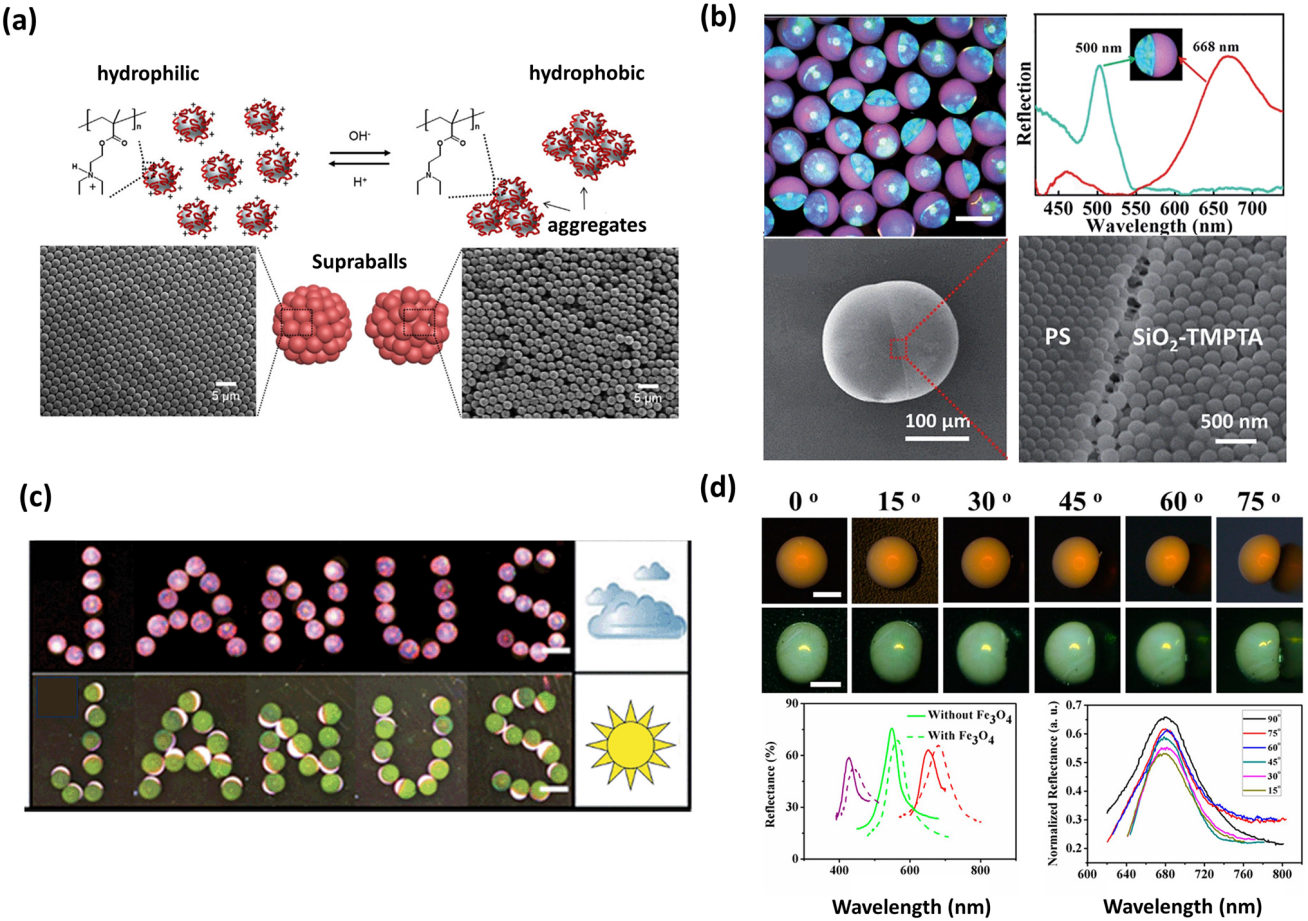
Supraparticles with colloidal photonic crystals structure. **a** The highly monodispersed NPs produced in an acidic environment showing order arrangement and causing the formation of a photonic crystal structure, while an agglomeration of NPs formed in an alkaline environment resulting in an amorphous structure. Reproduced with permission from Ref. [102], Copyright 2017, American Chemical Society. **b** Optical image, reflection spectra, and SEM images of PS/SiO_2_ Janus supraparticles with dual photonic bandgaps. Reproduced with permission from Ref. [107], Copyright 2014, The Royal Society of Chemistry. **c** PS/Fe_3_O_4_ Janus supraparticles with dual photonic bandgaps, in which the superparamagnetic Fe_3_O_4_ chains run perpendicular to the equator interface. The PS/Fe_3_O_4_ Janus supraparticles switch aimed at different light intensities, where PS hemispheres are upside under weak light intensity and Fe_3_O_4_–TMPTA hemispheres are upside under strong light intensity, making them ideal candidates for color conversion devices, as well as day and night dual-viewed displays. Reproduced with permission from Ref. [96], Copyright 2014, The Royal Society of Chemistry. **d** Optical microscopic images and reflectance spectra of microbead- and micro-ellipsoid-shaped supraparticles with Fe_3_O_4_ NPs incorporation at various view angles, as well as the reflectance spectra of the supraparticles with and without Fe_3_O_4_ NPs incorporation. Reproduced with permission from Ref. [119]. Copyright 2015, American Chemical Society

Colloidal photonic crystals are one kind of photonic crystals formed by the ordered arrangement of materials with different refractive indices [124–128]. Especially, these crystals consist of ordered self-assembled colloidal NPs [129–136]. Indeed, a supraparticle prepared using the SHS-assisted droplet template evaporation strategy can possess a photonic crystal structure, if the number of NPs inside the droplet is sufficiently large to induce early clustering of NPs [71, 137]. The critical number of NPs in the system generally depends on the parameters affecting the solvent evaporation rate and the diffusivity of NPs. Hence, the photonic crystal structure can be realized via precise control of the humidity and temperature throughout the evaporation, as well as the original NP suspension properties. Recently, it has been considered that the mono-dispersity of NPs has a significant effect on the formation of photonic crystal structures [102, 106]. Hans-Jürgen Butt’s group reported a supraparticle composed of pH-responsive NPs whose packing structure can be tuned by the mono-dispersity of NPs [102]. In this system, highly monodispersed NPs caused by a low pH environment showed order arrangement, resulting in a formation of the photonic crystal structure. On the contrary, a high pH environment caused an agglomeration of pH-responsive NPs, thus forming an amorphous structure (Fig. 7a) [102].

The supraparticles endowed with photonic crystal structure can exhibit unique photonic bandgap properties and bright structural colors [138–141], and they can be shaped into various forms, such as microwells, micro-balls, micro-ellipsoidal and micro-doughnut by regulating the dynamic behavior of TPCL during evaporation [103]. These features make them great application prospects in the field of converters, decorative coatings, displays, optical switching, and pigments. For example, Roman Krahne’s group reported a hollow-dome-shaped supraparticle with a photonic crystal structure produced by the evaporation-induced self-assembly process of CdSe/CdS nanorods onto SHS [113]. This photonic crystal structure showed tremendous prospects in the field of color converters, as it can be freely positioned in an excitation beam [113]. Orlin D. Velev’s group developed a supraparticle with concentric color rings, which were created from a mixed suspension of latex microspheres and gold NPs [106]. By placing droplets of the mixed suspension onto SHS and allowing slow evaporation, the latex particles formed a face-centered cubic (FCC) lattice, while most of the small gold NPs transferred to the supraparticle surface. This arrangement produced localized photonic crystal domains and resulted in the appearance of various color rings, offering potential applications in decorative coatings [106]. Additionally, the supraparticles composed of dual photonic crystal structures have been fabricated by governing the surface tensions between the two immiscible components via a tri-phase microfluidic device, where each part was composed of self-assembled NPs. When NPs employed in the system show different compositions and sizes, two distinct structural colors and reflection peaks can be found from these supraparticles (Fig. 7b) [107]. Among all dual-bandgap supraparticles, the PS/Fe_3_O_4_ Janus supraparticle can be considered as a particular one. Since the Fe_3_O_4_ hemisphere with green structural color can roll over when applying a magnetic field, the interchange of the top positions of the red PS and green Fe_3_O_4_ hemispheres can be easily achieved by altering the magnetic field's direction. This dynamic color conversion property renders it a promising candidate for optical switching materials, as well as for the creation of day-night dual-viewed bead panels (Fig. 7c) [107]. Zhou’s group reported a kind of supraparticle with non-iridescent structural colors and special microscopic shapes (e.g., microwells, dimpled microbeads, microbeads or micro-ellipsoidal) fabricated by evaporation-induced self-assembly of colloidal NPs on SHS [119]. In order to enhance the coherent structural color and eliminate the strong incoherent light-scattering, smaller-sized Fe_3_O_4_ NPs as light absorbers were added into this system. The microbead- and micro-ellipsoid-shaped supraparticles with Fe_3_O_4_ NPs incorporation showed bright structural colors and sharp reflection peaks, and the structural colors and reflection peak positions remained almost unchanged with the modification of observed angles from 0 to 75° (Fig. 7d). The structural color independent of angles can be attributed to the distinctive microstructure, which comprises a polycrystalline surface layer and an amorphous inner layer. Moreover, the polycrystalline structure is composed of “crystalline regions” with FCC structure and ‘‘amorphous regions’’ with randomly arranged NPs. These non-iridescent supraparticles with controlled micro-shapes have promising applications in the fields of nontoxic, nonbleaching pigments and energy-efficient full-color display pixels [119].

The employment of the SHS-assisted droplet template evaporation method for fabricating colloidal photonic crystals offers numerous advantages. This technique enables the fabrication of colloidal photonic crystals with diverse and flexible shapes, as well as integrated functionalities. However, it is important to acknowledge that the strict formation conditions may pose potential drawbacks to mass manufacturing.

### Catalysts

There are significant advantages to applying supraparticles in the catalysts field. Firstly, the inherent mesoporous structures of supraparticles make them possess large surface areas, offering huge benefits for enhancing catalytic activity. Moreover, supraparticle catalysts generally show better stability compared to individual NPs. Furthermore, by utilizing functionalized NPs and controlling suspension properties, supraparticles can be tailored to possess specific shapes, distinct structures, and emerging or synergetic properties, which offers the possibility for their use in specific catalytic reactions. As typical examples, the recent advancements in the utilization of supraparticles prepared by SHS-assisted droplet template evaporation strategy for multi-type catalysis, including photocatalysis, electrocatalysts, and enzyme catalysis, will be discussed.

**Fig. 8:**
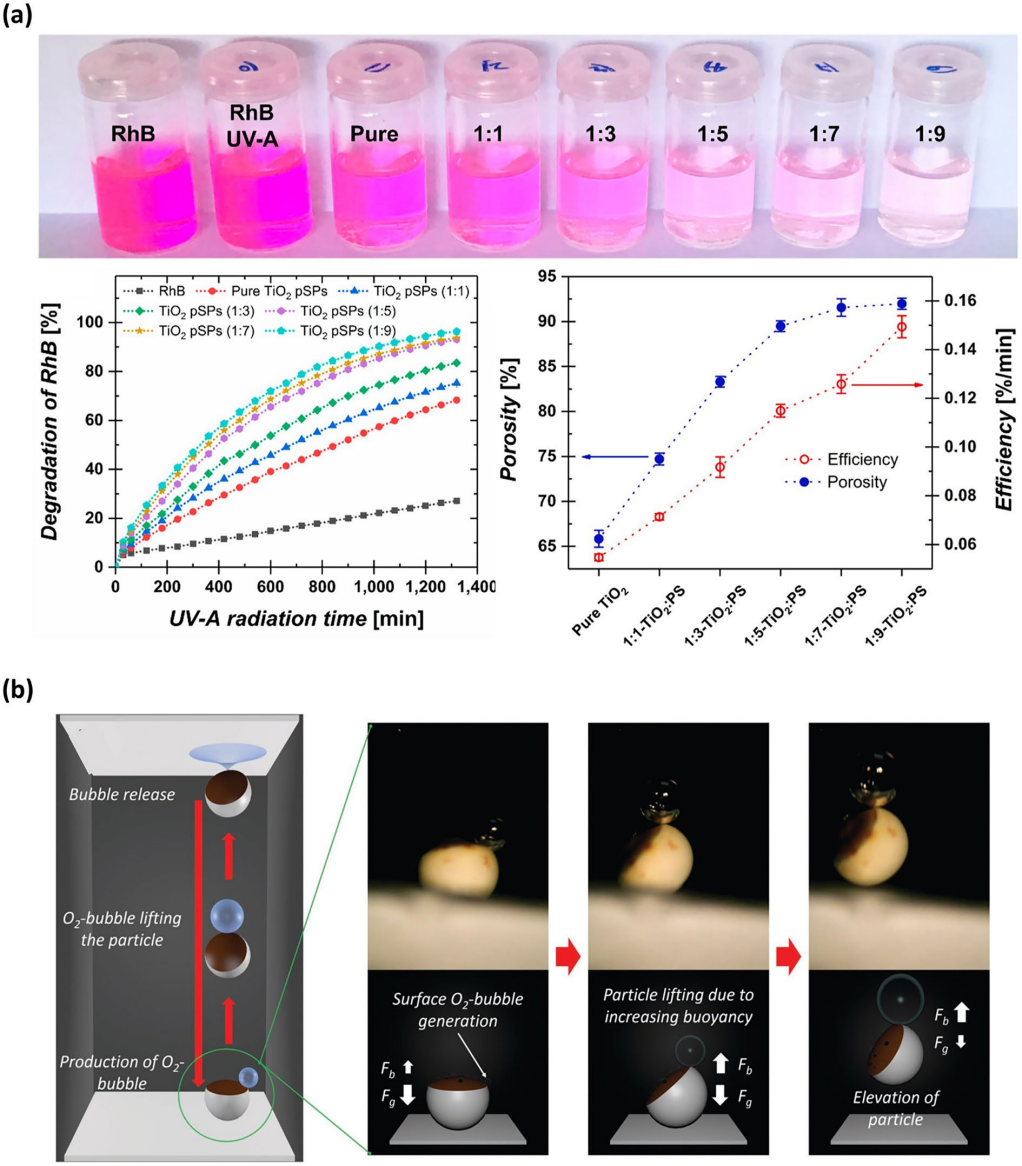
Supraparticles fabricated by SHS-assisted droplet template evaporation applied in the field of catalysts. **a** TiO_2_ supraparticles with varied porosity used as photocatalysts for RhB photodegradation. The ratios in the top figures represent the volume ratio of TiO_2_ to PS in the initial droplet of the mixed suspension, which is 1:1, 1:3, 1:5, 1:7, and 1:9, respectively. Reproduced with permission from Ref. [105], Copyright 2019, American Chemical Society. **b** The patchy particles composed of Pt-covered Fe_3_O_4_ NPs patches and silica framework used as electrocatalysts for H_2_O_2_ decomposition. The O_2_ bubble formed through H_2_O_2_ decomposition can lift the particle, facilitating the self-propelling motion of supraparticles. Reproduced with permission from Ref. [146], Copyright 2016, WILEY–VCH

Mesoporous semiconductor materials, owing to their wide bandgaps, have demonstrated remarkable performance in the field of photocatalysis research [142, 143]. For example, Hans-Jürgen Butt and co-authors reported TiO_2_ supraparticle photocatalysis with controlled porosity fabricated by firstly obtaining TiO_2_-PS composite supraparticles via SHS-assisted droplet template evaporation strategy and then removing the PS microspheres by calcination [105]. These TiO_2_ supraparticle catalysts showed porosity-dependent photocatalytic efficiency, as confirmed by the photodegradation experiment of rhodamine B (RhB) (Fig. 8a). The aqueous solution containing TiO_2_ supraparticles with the largest porosity demonstrated the highest level of degradation (∼97%) under UV-A light irradiation (power: 0.3 ± 0.01 mW cm^−2^, irradiation time: 22 h), resulting in almost complete bleaching of the RhB solution [105]. The enhancement in photocatalytic activity with increasing porosity can be attributed to the fact that the presence of macropores promoted the diffusion of organic dyes into supraparticles. In recent developments, alternative oxide materials, such as ZnO, SnO_2_, and Nb_2_O_5_, have gained attention as ideal substitutes for TiO_2_, since they made a breakthrough in overcoming the lower electron mobility of TiO_2_ material [144]. Hans-Jürgen Butt’s group proposed a simple strategy to prepare mesoporous ZnO and SnO_2_ supraparticles by evaporating homologous NP dispersions onto SHS [67]. In addition, this group also reported the coupled ZnO/TiO_2_ and TiO_2_ /SnO_2_ composite supraparticles fabricated through a sequential drying process of NP dispersions onto SHS [67]. Compared with pure metallic oxides, the coupled metallic oxide composite demonstrated a largely improved photocatalytic activity, as it can promote efficient spatial separation of electrons and holes [144, 145].

Currently, Pt and its alloys are recognized as the best electrocatalysts for oxygen reduction reactions. However, the high cost of Pt materials and declining activity have greatly hindered their large-scale use [142]. Integrating Pt into the supraparticle system by dint of SHS has become an effective approach to solving these problems. For instance, Michael Gradzielski and co-workers reported patchy supraparticles comprising Pt-covered Fe_3_O_4_ NPs as small patches embedded in a silica framework, which were fabricated by SHS-assisted droplet template evaporation strategy [146, 147]. Once these supraparticles were immersed in an aqueous H_2_O_2_ solution, a decomposition reaction of H_2_O_2_ occurred, resulting in the generation of oxygen bubbles in the solution (Fig. 8b). This observation indicated that the Pt-containing supraparticles exhibited high catalytic efficiency. Notably, these oxygen bubbles released at the air–water interface can cause an oscillating vertical motion and elevation of the supraparticle, thus making it an excellent self-propelling material [147]. However, the oxygen evolution in this system led to the supraparticles’ disintegration. To enhance their mechanical stability, the research group introduced a series of additives, such as PS micro-fibrillated cellulose or Na_2_SiO_3_ material, to reinforce the supraparticles [148].

The SHS-assisted droplet template evaporation strategy allows for the creation of supraparticles with complex properties through the aggregation of functionalized NPs. Thus, functionalizing NPs opens a novel avenue for preparing supraparticles with specific catalytic properties. One typical example is the immobilization of α-amylase, an enzyme catalyst designed for starch decomposition, into supraparticles using the covalent binding method [149]. The resulting enzyme catalyst, containing α-amylase, acts on the α-1,4-glycosidic bond between glucose units, leading to a sluggish liberating of maltose. This behavior further results in the loss of helical units in starch and hampers the formation of starch-I_3_^−^ complexes. Hence, when the enzyme catalysts were immersed in a medium containing iodine and starch, the medium underwent a color change from blue to transparent within a short period [149]. It indicates that starch realized a complete decomposition, demonstrating the excellent catalytic effect of this enzyme catalyst.

### Biomedicines

SHS offers a novel platform for processing spherical hydrogel microspheres. These microspheres hold significant potential in various biomedical fields, particularly in drug delivery and cell encapsulation. The remarkable superiorities of resulting hydrogel microspheres include (i) low production costs, as well as easy synthesis methods and mild processing conditions; (ii) nearly 100% encapsulation efficiency for various bioactive substances, such as cells and proteins; (iii) the possibility of achieving high drug load and a finely regulated drug release rate [150]; (iv) the realization of narrow size distribution and mass production [63, 74]; (V) wide adjustability of microsphere size, ranging from microns to centimeters [76].

**Fig. 9:**
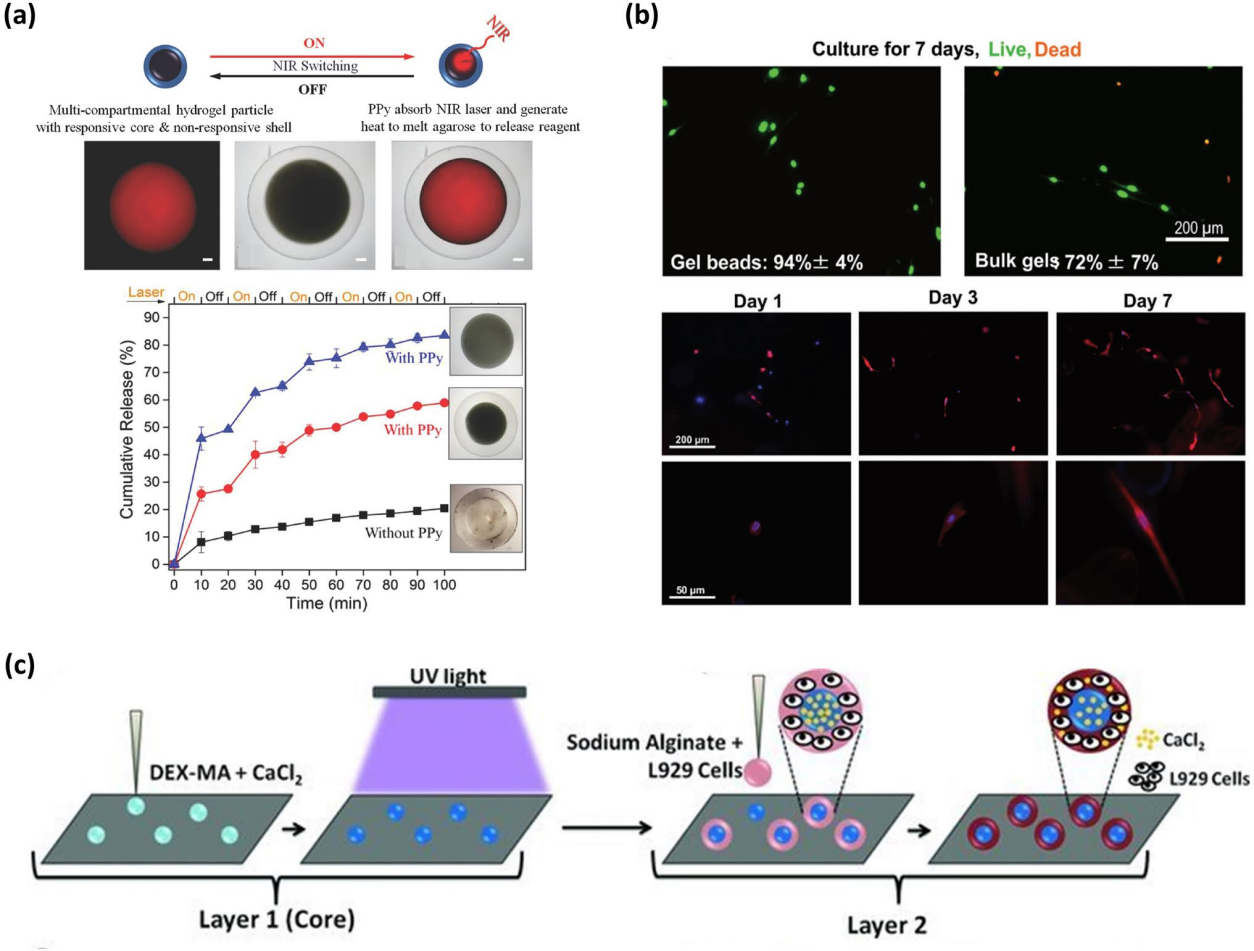
Hydrogel microspheres obtained by SHS-assisted cross-linking curing technique employed as drug delivery medium or cell encapsulation materials. **a** Release of TRITC-dextran drug triggered by NIR laser from a multi-compartment hydrogel microsphere with a core–shell structure. The core, loaded with polypyrrole NPs and TRITC-dextran, is sensitive to the NIR laser, while the non-loaded shell is not. A comparison of release rates of TRITC-dextran encapsulated in hydrogel microspheres is shown in a broken line chart. Reproduced with permission from Ref. [85], Copyright 2014, Wiley–VCH. **b** Viability test and observation of cell spreading phenomenon using MC3T3 mouse osteoblast cells. Reproduced with permission from Ref. [162], Copyright 2018, Wiley–VCH. **c** Schematic diagram illustrating the process of encapsulating L929 cells within the external layer of core–shell structured hydrogel microspheres. The core is formed through the cross-linking of DEX-MA under UV light, while the shell formation is attributed to the cross-linking of alginate induced by the release of Ca^2+^ immobilized in the inner layer. Reproduced with permission from Ref. [158], Copyright 2013, Wiley–VCH

The following is a detailed description of how microspheres have been utilized on SHS for drug delivery. Firstly, the target drugs are mixed with a monomer in an aqueous solvent. Secondly, the droplets of mixed solution are placed onto SHS and retain a spherical shape. When the monomer undergoes a cross-linking reaction, hydrogel microspheres are formed, along with drugs encapsulated in it. Finally, the release of drugs is achieved as the hydrogel microspheres exhibit swelling behavior in response to external stimuli. These stimuli typically include water [63], pH values [151], temperature [74], and light [85]. For instance, João F Mano’s group developed water-responsive alginate hydrogel microspheres loaded with theophylline drug. The preparation involved an in-situ cross-linking process of alginate droplets and calcium chloride [63]. The resulting hydrogel microspheres exhibited a swelling behavior in response to water stimulus, allowing for the release of the alginate drug in a deionized water environment at 37 °C [63]. Their significant volume expansion in water provided more space for drug diffusion [152]. Consequently, the alginate hydrogel microsphere demonstrated a high release rate (several minutes) and remarkable release efficiency (almost 100%). Another study by this group involves pH-responsive chitosan hydrogel microspheres used as drug delivery materials. In this system, dexamethasone served as the drug model. The dexamethasone-loaded chitosan hydrogel microspheres were formed onto SHS via a neutralized reaction and an ionic gelation process [151]. The release profiles of dexamethasone indicated that the drug can be released at multiple pH values, with a little distinction observed across different pH levels [151]. This phenomenon can be attributed to the fact that the swelling degree of pH-responsive hydrogel microspheres is influenced by the pH value, but this system shows a relatively slow swelling rate. In many cases, it may take one or several days to observe a significant divergence in the swelling degree, whereas drug release typically concludes within a few hours [151]. Consequently, the release rate is primarily dependent on the diffusion of dexamethasone rather than pH values. Song and co-authors reported a composite hydrogel microsphere formed through the cross-linking process of the methacrylate-modified dextran and poly(N-isopropylacrylamide) (PNIPAM) [74]. The composite hydrogel microsphere exhibited temperature-responsive properties due to the low critical solution temperature of PNIPAM in the aqueous medium (around 32 °C) [153–155]. When loaded with proteins such as bovine serum albumin or insulin, the composite hydrogel microsphere showed a fast release of the protein below 32 °C, and the release rate could be regulated by adjusting the proportion of microsphere constituents and the temperature of the medium. These findings provide evidence for the feasibility of utilizing hydrogel microspheres to develop controlled drug/protein delivery systems [74]. Although the above spherical hydrogel microspheres exhibited many merits in the drug storage and release system, their single-compartmental structures make them lack combinational functionalities, such as the ability to control the material properties independently or achieve synergetic therapeutic effects [156, 157]. To overcome this shortcoming, a novel multi-compartmental hydrogel has been employed in drug delivery [85, 86, 158]. For instance, Chia-Hung Chen’s group fabricated a core–shell structured multi-compartment hydrogel microsphere with selective near-infrared (NIR) light sensitivity [85]. The core compartment was composed of agarose/alginate hydrogel microspheres loaded with polypyrrole NPs and dextran drug, while the non-loaded agarose/alginate hydrogel served as the shell (Fig. 9a) [85]. The core compartment, due to the high photothermal conversion effectiveness of polypyrrole NPs [159–161], exhibited NIR responsiveness, resulting in a release of the dextran drug, while the non-loaded shell served as a barrier, controlling the drug release. Thus, this multi-compartment structure enables individual control of the release speed. In addition to the capability of independently regulating the release speed of individual compartments, multi-compartment systems have the potential to deliver multiple molecules simultaneously. João F. Mano’s group reported a multilayered hydrogel microsphere prepared by a sequential-crosslinking process on SHS, in which every layer was loaded with different molecules, thus creating a possibility of realizing the sequential release of various molecules [158]. Therefore, this multilayered hydrogel microsphere may exhibit promising prospects for the synergistic treatment of multi-factorial diseases.

Cell encapsulation is a strategy to encapsulate a pool of living cells within a semipermeable membrane or hydrogel. The utilization of the SHS-assisted cross-linking technique has been regarded as a highly productive approach for encapsulating viable cells within hydrogel materials [76]. As a typical example, the procedure for encapsulating MC3T3 mouse osteoblast inside the hydrogel microsphere will be described in detail [162]. This encapsulating hydrogel material was obtained from a cross-linking process of 4-arm polyethylene glycol (PEG)-vinylsulfone and degradable metalloproteinase, which both were dissolved in the spherical droplets onto SHS. To ascertain the viability of cell encapsulation, MC3T3 mouse osteoblast cells were encapsulated and cultured in a controlled environment (CO_2_ incubator, pH 7.4, 37 °C), followed by a staining experiment. The live/dead staining result showed that cell viability exceeded 94% after 7 days of cultivation (Fig. 9b). Furthermore, the cell spreading results monitored by fluorescent microscopy confirmed the sustained healthy growth of the encapsulated cells for 7 days. Subsequently, most of the cells were released onto a tissue culture polystyrene Petri after 14 days due to the degradation of the sensitive metalloproteinase network. The findings demonstrate that the SHS-assisted cross-linking curing methodology is a highly feasible approach to creating hydrogel microspheres for encapsulating living cells. Undoubtedly, other types of cells can be effectively encapsulated within specific hydrogel microspheres. For instance, João F. Mano’s group reported the encapsulation of L929 cells within alginate hydrogel microspheres, which involved the deposition of an alginate suspension containing the cells onto SHS, followed by the cross-linking of alginate with Ca^2+^ ions [63]. The study revealed that the L929 cells exhibited a homogeneous distribution within the hydrogel microspheres, indicating that the employed technique enables the creation of cell-encapsulating hydrogel microspheres with uniformity. Besides these single-compartmental microspheres, this processing strategy is also suitable for constructing multi-compartment structures. By loading each compartment with an active agent or cell, the multi-compartment structures exhibit great application prospects in the encapsulation of multiple substances and cell therapy. For instance, João F. Mano’s group reported a bi-layered hydrogel microsphere loading L929 cell in the outer layer and CaCl_2_ molecule in the inner layer [158]. In this system, the core layer consisted of a dextran modified with methacrylic groups (DEX-MA) hydrogel, while the shell was composed of alginate hydrogel. The hardening of the core layer was achieved through the UV-light-induced cross-linking of DEX-MA, whereas the formation of the alginate shell was attributed to the cross-linking of alginate induced by the diffusion of Ca^2+^ ions from the core (Fig. 9c) [158]. Cell viability tests confirmed that L929 cells encapsulated in this structure can maintain viability for 72 h and exhibited a homogeneous distribution on the shell layer [158], which can be attributed to the fact that this mild SHS-assisted cross-linking processing methodology significantly reduced an invasion of cell encapsulation and protected the viability of loaded cells [163]. Furthermore, this configuration holds potential for specific therapeutic applications, as the inner layer of the microsphere can serve as a reservoir for bioactive or therapeutic agents that influence cell behavior. Additionally, the composition of the compartmentalized microsphere can be easily tailored by adjusting the formulation of the NP suspensions deposited on the SHS, thereby meeting the requirements for encapsulating different cell types or bioactive factors.

### Driven Crystallization

Evaporating solvents of droplets on SHS triggers a concentration convergence effect within a confined region, resulting in the formation of microsphere-like materials with distinct shapes and crystalline structures. A comprehensive description of the formation process can be found in Sect. 2.3 (Fig. 3a).

**Fig. 10:**
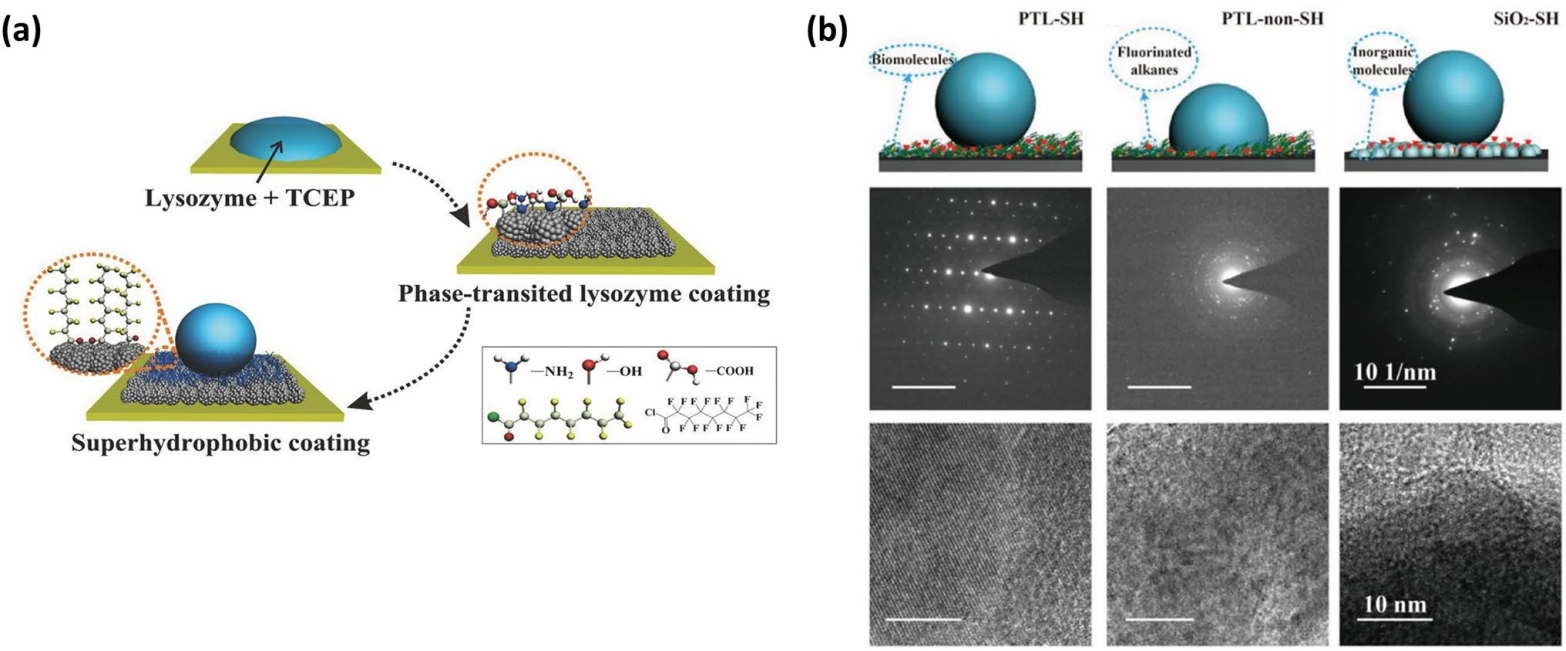
SHS-assisted droplet template evaporation strategy used to drive protein crystallization. **a** Schematic illustration of the formation of phase-transited lysozyme surface with superhydrophobicity. Reproduced with permission from Ref. [97]. Copyright 2015, Wiley–VCH. **b** Scheme diagrams (top), Selected area electron diffraction diagrams (middle) and high-resolution transmission electron microscope diagrams (bottom) of lysozyme crystals formed on different surfaces, where PTL-SH, PTL-non-SH and PTL-non-SH represent the phase-transited lysozyme surface with superhydrophobicity, phase-transited lysozyme surface without superhydrophobicity, and SiO_2_ surface with superhydrophobicity, respectively. These images demonstrate that high-quality crystals are only formed on a bio-based SHS. Reproduced with permission from Ref. [96]. Copyright 2018, Wiley–VCH

Nowadays, the SHS has become a common tool to induce the crystallization of various substances, including proteins [97], peptides [96] and salts [164]. To illustrate this phenomenon, we will describe the crystallization process of lysozyme protein as reported by Yang’s group [97]. Initially, the sessile droplet of lysozyme buffer was deposited onto the protein-based SHS, which was fabricated by assembling phase-transited lysozyme product into a rough structure and then coating low surface energy materials onto it (Fig. 10a). Due to the high-water repellency of the SHS, the droplet stood onto SHS as a spherical shape, resulting in an ultra-small liquid/solid contact area. As the solvent within the droplet gradually evaporated, a concentration convergence effect occurred within the limited contact area, thereby facilitating the formation of a protein crystal nucleus [97, 111]. Subsequently, the crystal nucleus grew over time, eventually causing the formation of a protein crystal with a large size. Specifically, when the volume of the droplet onto SHS was set to 10 µL, the crystal nucleus appeared after a 1.5-h evaporation process, and the crystallization process was nearly complete within another hour, ultimately yielding the lysozyme crystal exceeding 100 µm in size [97]. The growth of lysozyme crystal followed a classical crystallization theory and the crystalline domains appeared in two forms known as hexagonal and tetragonal. It is worth mentioning that the lysozyme protein can achieve high-quality crystallization at a fast rate (within several hours) even under a low protein concentration (≈70 × 10^−6^ mol L^−1^) [97]. In comparison to traditional methods that require several days and higher protein concentrations for successful crystallization, this process significantly accelerates the crystallization speed while reducing the required protein concentration [97]. In addition, this manufacturing process avoids the use of toxic additives, complicated crystallization formulas, and harsh crystallization conditions. Consequently, this SHS-assisted droplet template evaporation technique proves to be an efficient and cost-effective approach for facilitating protein crystallization.

The SHS-assisted droplet template evaporation strategy holds particular significance for the crystallization of the protein whose source is comparatively rare. For instance, a seven residue, fibril-forming peptide with sequence GNNQQNY, β-ketoacylacyl carrier protein synthase III [165], that is traditionally challenging to crystallize, can realize a high-quality crystallization onto bio-based SHS, as reported by Yang’s group [96]. In addition, through further observing the crystallization behavior of proteins on three different surfaces, including bio-based SHS, superhydrophobicity and non-bio-based SHS, it has been discovered that the high-quality protein or polypeptide crystals only existed on a bio-based SHS (Fig. 10b) [96]. This can be attributed to the fact that bio-based SHS not only possesses micro/nanostructures that can facilitate solute concentration, but also incorporates numerous functional chemical groups (e.g., methyl, thiol, amino) that provide abundant active nucleation sites to enhance biomolecular crystal growth [96, 166]. Due to these favorable characteristics, bio-based SHS can expedite the crystallization process of various substances, including proteins, polypeptides, and salts, even at low solute concentrations. Thus, it is highly desirable for large-scale production of target material crystallization. Furthermore, by introducing combinatorial chemistry into the target material intended for crystallization, the interactions between the crystals (e.g., specialized proteins) and drugs can play a significant role in biomedical and therapeutic applications, whether in vivo or in vitro.

### Trace Analyte Detection

**Fig. 11:**
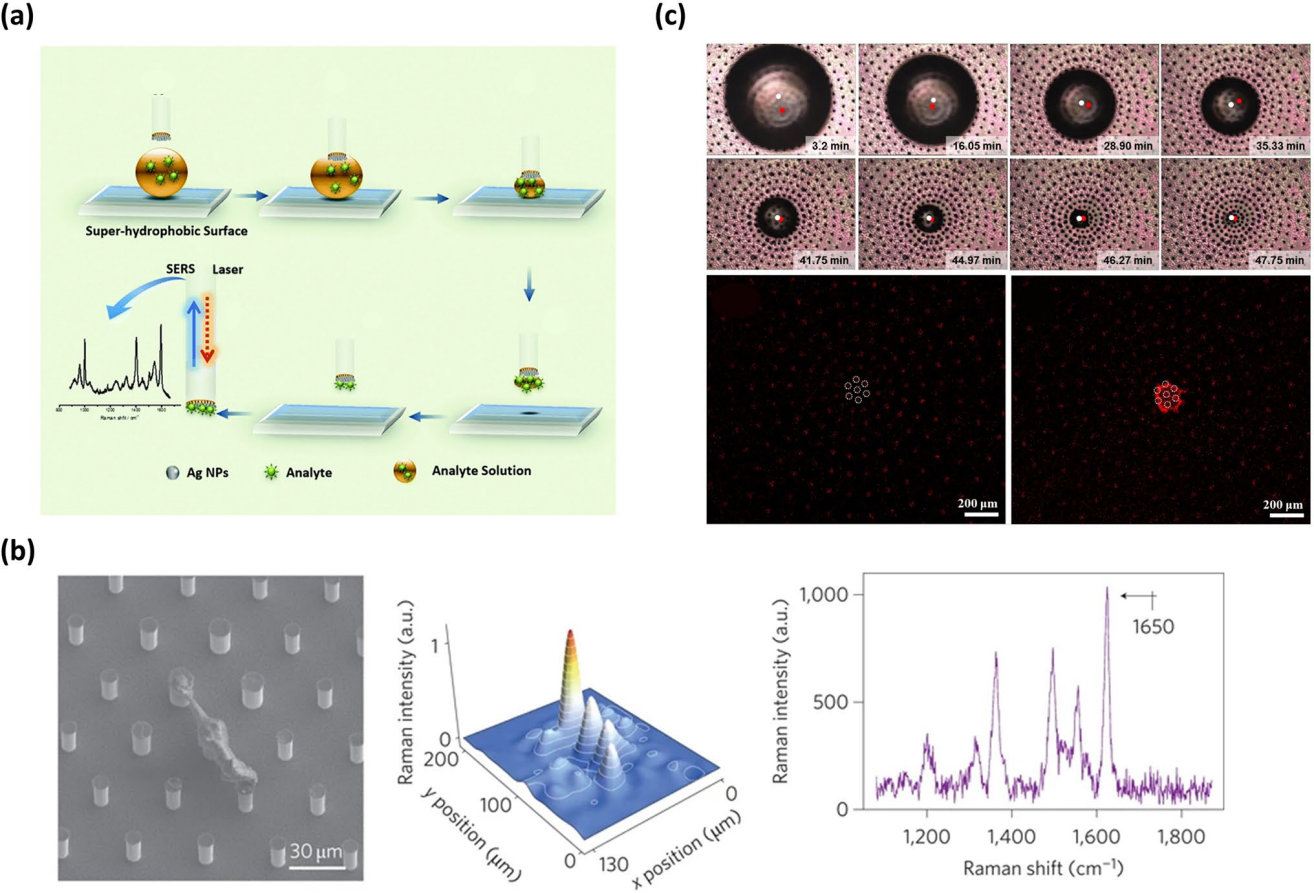
SHS-assisted droplet template evaporation strategy employed to induce a solute concentration and its application in trace analyte detection. **a** Schematic diagram illustrating the process of solute concentration onto the SHS and SERS analysis of the solute. Reproduced with permission from Ref. [171]. Copyright 2015, The Royal Society of Chemistry. **b** SEM image, Raman mapping measurement, and spectral signature of rhodamine solute precipitation from a 10^–17^ mol·L^−1^ solution onto a dual-functional structure comprising silicon micropillar arrays adorned with silver NPs on the top. Reproduced with permission from Ref. [173]. Copyright 2011, Nature Portfolio. **c** The still-shot images and confocal laser scanning microscope images showing the evaporation process of droplets and the aggregation process of solute onto the structure composed of the micropillar array with a radial density gradient. Reproduced with permission from Ref. [174]. Copyright 2017, American Chemical Society

The concentration convergence effect induced by evaporating aqueous droplets onto SHS is also highly significant in molecular analysis and detection. This is because the detection thresholds and detection sensitivities of existing spectral analytical methods heavily rely on the solute concentrations [167–169]. In this section, Raman scattering (SERS) spectroscopy serves as an illustrative example to demonstrate the influence of solute concentration [170]. When SERS spectroscopy is employed for qualitative and quantitative detection of a target solute, the SERS signals of a small number of analyte molecules falling into a “hot spot” occupy a high proportion of SERS signals in the whole measurement. Hence, it is a feasible method that preconcentrating the solute molecules onto SHS increases the probability of the analyte molecules entering the hot spot. Alexandre G. Brolo’s group elucidated the process of SERS analysis with the help of SHS (Fig. 11a) [171]. Firstly, a droplet of an aqueous solute solution was deposited on the SHS, maintaining a spherical shape. Subsequently, the SERS optrode was positioned at the top of the droplet. As the solvent evaporated, the droplet size continually decreased and the contractive droplet was completely captured by the hydrophilic tip of the SERS optrode. Finally, the solute achieved enrichment at the hydrophilic tip, enabling its easy detection through a SERS analysis [171]. With the utilization of this device, the lowest experimentally detected amount of nile blue A was 25 fg (34 attomoles). Moreover, triazophos, an organophosphate pesticide utilized in agriculture, can be detected in quantities as low as 20 pg (64 femtomoles). In addition to the configuration where SHS only serves as a medium to deliver concentrated analytes to the SERS substrate, the dual-functional SHS, which not only concentrates solute but also provides SERS hot spots, has also been developed [172, 173]. For instance, E. Di Fabrizio and colleagues reported the fabrication of multi-types of dual-functional SHS by combining silicon micropillar arrays with plasmonic nanostructures [173]. Among the multi-types of SHS, the simplest structure consisting of silicon micropillar arrays topped with silver NPs was employed to demonstrate how to achieve a low detection threshold and high detection sensitivity. In this system, the rhodamine 6G served as a typical solute model. Initially, when the solvent of the droplet containing rhodamine 6G evaporated on the bi-functional substrate, the TPCL moved freely, causing a decrease in droplet size while maintaining a constant droplet shape. As the evaporation progressed, the solution became more concentrated. Upon the culmination of the process of evaporation, wherein the shape and concentration attained a state of instability, the droplet underwent a collapse, leading to the deposition of rhodamine 6G in a specific region. Since the precipitation regions and the “hot spots” areas of this bi-functional substrate were coincident, rhodamine 6G can be detected even at concentrations as low as 10^–17^ mol L^−1^. Micro-Raman mapping measurement and spectral analysis confirmed the presence of solute rhodamine 6G through a clear band center at 1,650 cm^−1^ (Fig. 11b) [173]. Furthermore, by combining this structure with a fluorescence device, DNA can be detected at concentrations as low as 10^–18^ mol L^−1^ [173].

While the above dual-functional structure can enhance detection sensitivity, there is an inevitable migration of droplets onto SHS during the evaporation process, leading to uncertain deposition sites. Hence, when performing SERS analysis, the surface should be completely examined unless the target is fluorescently labeled. To address this issue, Shin-Hyun Kim and colleagues proposed a structure with uneven density [174]. This structure was composed of the micropillar array with a radial density gradient, where the nanotip array was located on the top surface of each micropillar and Ag NPs were deposited on the nanotips. Since a radial density gradient of micropillar causes a radial gradient of contact angle onto the structure surface, the droplets can spontaneously move towards the central region with the highest density [174]. Consequently, the analyte dissolved in the droplets can be enriched at the central surface of the structure during the evaporation process (Fig. 11c) [174]. The analyte can be detected at predetermined positions using Raman spectra without requiring a complete scan of the substrate, thus significantly saving the detection time [174]. This makes it a general structure to promote the enrichment of the solute [175].

In summary, the SHS-assisted droplet template evaporation strategy plays a significant role in molecular detection, particularly for molecules with concentrations as low as the attomolar scale. Additionally, this approach has the potential to be integrated with various spectral analysis techniques (e.g., SERS spectra, fluorescence, and Raman), and is fully compatible with existing biological and medical protocols. Consequently, it is of great significance for the analysis of rare or hazardous chemicals involved in biomedical, food safety and ecological pollution.

## Summary and Future Perspective

In this review, we have presented a comprehensive overview of the recent advancements in the field of SHS-assisted preparation of microspheres and supraparticles, as well as their applications. Firstly, the strategies for fabricating microspheres and supraparticles, including SHS-assisted cross-linking curing, SHS-assisted polymer melting, and SHS-assisted droplet template evaporation, have been presented. Especially, the preparation processes of supraparticles have been described in detail in terms of morphology, structure and properties. In addition, we also summarized the influence of the dynamic TPCL behavior of droplets during the evaporation process on the resulting shapes of supraparticles. Then, we demonstrated the wide range of applications for microspheres and supraparticles fabricated through the SHS-assisted strategies. These applications encompass advanced optical devices, catalysts with superior catalytic efficiency, drug delivery systems with controlled release rates, cell encapsulation materials with improved encapsulation efficiency, Polymer crystallization, and molecular trace detection.

Although significant progress has been made, there are still challenges and potential outcomes that need to be addressed. These difficulties and potential outcomes are outlined below:

1. Size and Shape Control: Achieving precise control over the size and shape of microspheres and supraparticles remains a challenge. While SHS-assisted methods offer some level of control, further optimization is needed to achieve mono-dispersity and uniformity in size and shape. Exploring the impact of original droplet size and solute concentration on the resulting particle size, as well as conducting systematic investigations into the factors influencing different supraparticle morphologies, will offer significant benefits for fine-tuning fabrication parameters in SHS-assisted methods. This exploration will lead to improved control over size and shape, ultimately enhancing reproducibility in the process.
2. Scalability and Production Efficiency: Scaling up the production of microspheres and supraparticles while maintaining their desired properties is an ongoing challenge in the field. SHS-assisted methods often involve complex fabrication processes that can be time-consuming and require specialized equipment to ensure uniform manufacturing. Additionally, these specialized equipment often have limited throughput, typically ranging from 100 to 1,000 droplets per second [64]. Developing strategies to improve production efficiency, such as modifying microfluidic devices by integrating multiple channels and sprinklers, may be expected to enable the fast fabrication of microdroplets.
3. Reproducibility, Stability, and Durability: Ensuring the good reproducibility of the SHS-assisted methods, the long-term stability and durability of microspheres and supraparticles is essential for their practical applications. The reproducibility of the SHS-assisted methods mainly depends on the durability of SHS. Thus, investigating the impacts of various nano-microstructures and modification techniques on the durability of SHS, with the aim of finding a robust SHS, assumes significant importance in maintaining consistent production of supraparticles and microspheres. Concerning the stability and durability of microspheres and supraparticles, multiple factors come into play, including structural integrity, resistance to degradation, and stability under various environmental conditions. Exploring the factors holds promise for advancing the practical utility of microspheres and supraparticles.
4. Functionalization and Integration: Expanding the functionality and integration of microspheres and supraparticles is a promising direction for future research. SHS-assisted methods can potentially enable the incorporation of functional elements or materials within the microspheres or on their surfaces. Developing strategies for precise functionalization and integration will enhance their capabilities and enable new applications. One example is the combination of catalytic performance with controlled self-propelled motion features, which holds significant potential across various domains, including oil cleanup, pollutant decomposition, agitation and mixing, and selective chemical reactions. Another area of interest is the development of multiple-compartment microspheres that enable the sequential release of multiple drugs. Although multi-compartment structures and drug delivery systems with controlled release rates have been developed, the diffusion of drugs between chambers and the external environment remains an unavoidable issue, which leads to the failure of sequential delivery. To address this problem, it is important to select drug delivery systems with large release rate differences. The integration of these systems into a single drug delivery system holds significant potential for achieving sequential drug release. In summary, the functionalization and integration of microspheres and supraparticles offer exciting prospects for future research.
5. Characterization and Understanding: Comprehensive characterization techniques and an in-depth understanding of the formation mechanisms are essential for further advancements in microspheres and supraparticles fabricated by SHS-assisted methods. Efforts should be made to develop advanced characterization techniques that provide detailed information about their structural, optical, mechanical, and chemical properties. This knowledge will contribute to the optimization of fabrication processes and the development of tailored microspheres and supraparticles.

By addressing these challenges and exploring their potential outcomes, further advancements can be achieved in the SHS-assisted preparations of microspheres and supraparticles, as well as their applications. Overcoming these challenges will bring new opportunities for their use in various fields, including materials science, biotechnology, medicine, and environmental applications.
